# Supplementary material for: Towards an interoperable perovskite description or how to keep track of 300 perovskite ions
Source: Nat Commun. 2025 Sep 30;16:8725. doi: 10.1038/s41467-025-64325-x (PMC12484728; doi:10.1038/s41467-025-64325-x)
Supplement: Supplementary file 1 — Supplementary Information [file 41467_2025_64325_MOESM1_ESM.pdf]

Supplementary Information

Towards an Interoperable Perovskite Description

Or How to Keep Track of 300 Perovskite Ions

Ayman Maqsood[1], Hampus Näsström[2], Chen Chen[1], Li Qiutong[1], Jingshan Luo[1], Rayan Chakraborty[3], Volker Blum[2], Eva Unger[4,5], Claudia Draxl[2], José A Márquez*[2], T. Jesper Jacobsson*[6]

1. Institute of Photoelectronic Thin Film Devices and Technology, State Key Laboratory of Photovoltaic Materials and Cells, Tianjin Key Laboratory of Efficient Utilization of Solar Energy, Ministry of Education Engineering Research Center of Thin Film Photoelectronic Technology. Nankai University, Tianjin 300350, China
2. Department of Physics, Humboldt-Universität zu Berlin, Berlin, Germany
3. Thomas Lord Department of Mechanical Engineering and Materials Science, Duke University, Durham, NC, United States of America
4. Helmholtz-Zentrum Berlin für Materialien und Energie GmbH, HySPRINT Innovation Lab: Hybrid Materials Formation and Scaling, Kekuléstraße 5,12489, Berlin, Germany
5. Division of Chemical Physics and Nano Lund, Lund University, Box 124, 22100 Lund, Sweden
6. Department of Physics, Chemistry and Biology (IFM), Linköping University, Linköping, Sweden

Corresponding authors:
T. Jesper. Jacobsson. jacobsson.jesper.work@gmail.com, jesper.jacobsson@liu.se
José A Márquez. josemarquez@physik.hu-berlin.de

Supplementary Notes

Description of the JSON Schema

A description of all the elements suggested for describing the perovskite composition is listed below.

long_form

The perovskite composition according to IUPAC recommendations, where standard abbreviations are used for all ions. A-site ions are listed in alphabetic order, followed by the B-site ions in alphabetics order, followed by the X-site ions in alphabetic order, all with their stochiometric coefficients. For increased clarity, we recommend enclosing all ions whose abbreviations are 3 letters or longer in parentheses. Example: "$Cs_{0.05}FA_{0.78}MA_{0.17}PbBr_{0.5}I_{2.5}$".

short_form

The *long_form* stripped of the numeric coefficients. This is a useful key for searching and groping perovskite data. Example. "CsFAMAPbBrI".

composition_estimation

A categorical description of how the composition is estimated. Standard options include: "Estimated from precursor solutions", "Literature value", "Estimated from XRD data", "Estimated from spectroscopic data", and "Theoretical simulation".

sample_type

A categorical description of the type of sample the data describes. Standard options include: "Polycrystalline film", "Single crystal", "Quantum dots", "Nano rods", and "Colloidal solution".

dimensionality

A categorical description of the dimensionality of the perovskite. Standard options include: "0D", "1D", "2D", "3D", "2D/3D". "2D/3D" refers to a situation cases where a 2D phase is intermixed with a 3D phase but where the overall composition is given by the value in *long_form*.

band_gap

The band gap of the perovskite expressed in electron volt. Details of how the band gap is estimated is suggested to be encapsulated in a separate data schema. A floating-point number.

ions_a_site

A list of dictionaries on the form [{key_1: value_1, key_2: value_2, …}, {key_1: value_1, …}, …]. Each element in the list represents one A-site ion in the perovskite structure. Each ion is given its own dictionary with the keys listed below.

ions_b_site

Same structure as the *a_ions*

ions_x_site

Same structure as the *a_ions*

abbreviation

The abbreviation used for the ion when writing the perovskite composition in the variable *long_form*. Examples. "Cs", "MA", "FA", "PEA"

coefficient
The stoichiometric coefficient of the ion. Implemented as a string such as “0.75”, or “1-x”. The rational for representing the coefficients as strings and not as floating-point numbers is to allow for situations where the coefficients are unknown.

molecular_formula
The molecular formula which indicates the numbers of each type of atom in a molecule, with no information about the structure. Examples. "Cs+", "CH5N2+", " C8H12N+", “Pb+2”

smiles
The canonical SMILES string of the ion. With this data, the organic ions can easily be visualised or used in computational software. Examples. “[Cs+]”, “C(=[NH2+])N”, “C1=CC=C(C=C1)CC[NH3+]”, “[Pb+2]”

common_name
The common or trivial name of the ion. It is common for ions to have more than one common name wherefor the data in this field could vary. Nevertheless, the trade name is worth reporting as this is the way it will be referred to in speech. Example. “Cesium ion”, “Formamidinium”, “Phenylethylammonium”, “Lead ion”

iupac_name
The preferred systematic IUPAC name of the ion. Example. "Cesium(1+)", "Formamidinium", “2-phenylethylazanium”, “lead(2+)”

cas_number
The CAS number for the ion. There are cases where CAS numbers not yet have been defined. Example. "18459-37-5", "17000-00-9"

source_compound_smiles
The smiles of the neutral parent or source compound. The source compound can vary and is thus not unique, but having data for a neutral source compound solves several problems. It deals with the ambiguity of the charge for diamines, which can be either +1 or +2, CAS numbers are more often defined for source compounds than for their ions, and it provides an entry point to what often is a commercially available starting material in the synthesis. For tertiary amines, there is no unique parent compound, but the I, Br, or Cl salts can often take that role. Example. "[Cs]", "C(=N)N", “C1=CC=C(C=C1)CCN”

source_compound_molecular_formula
The molecular formula for a neutral source compound.

source_compound_cas_number
The CAS-number for a neutral source compound.

source_compound_iupac_name
The systematic IUPAC name for a neutral source compound.

additives
A list of dictionaries on the form: [{key_1: value_1, key_2: value_2, …}, {key_1: value_1, …}, …]. Each element in the list represents a dictionary with properties for one additive present in the film but not incorporated into the perovskite crystal structure. Keywords in the dictionaries include: name, concentration (in /cm^3), mass_fraction, and data to uniquely identify the compound including the same key value pairs found in the sections for perovskite ions.

impurities
Has the same structure as the additives but refer to compounds that not intentionally have been added to the perovskite.

m_def
Should be "perovskite_solar_cell_database.composition.PerovskiteComposition". Has no direct meaning for the perovskite composition, but is required to aligning the files with the NOMAD database and for accessing additional NOMAD functionality.

NOMAD GUI

The landing page for the graphical user interface in NOMAD used for constructing perovskite composition files are given in figure S.1. Instructions for how to get to the GUI and for how to construct a perovskite composition is found online at: https://fairmat-nfdi.github.io/nomad-perovskite-solar-cells-database/how_to/create_a_perovskite_composition.html.

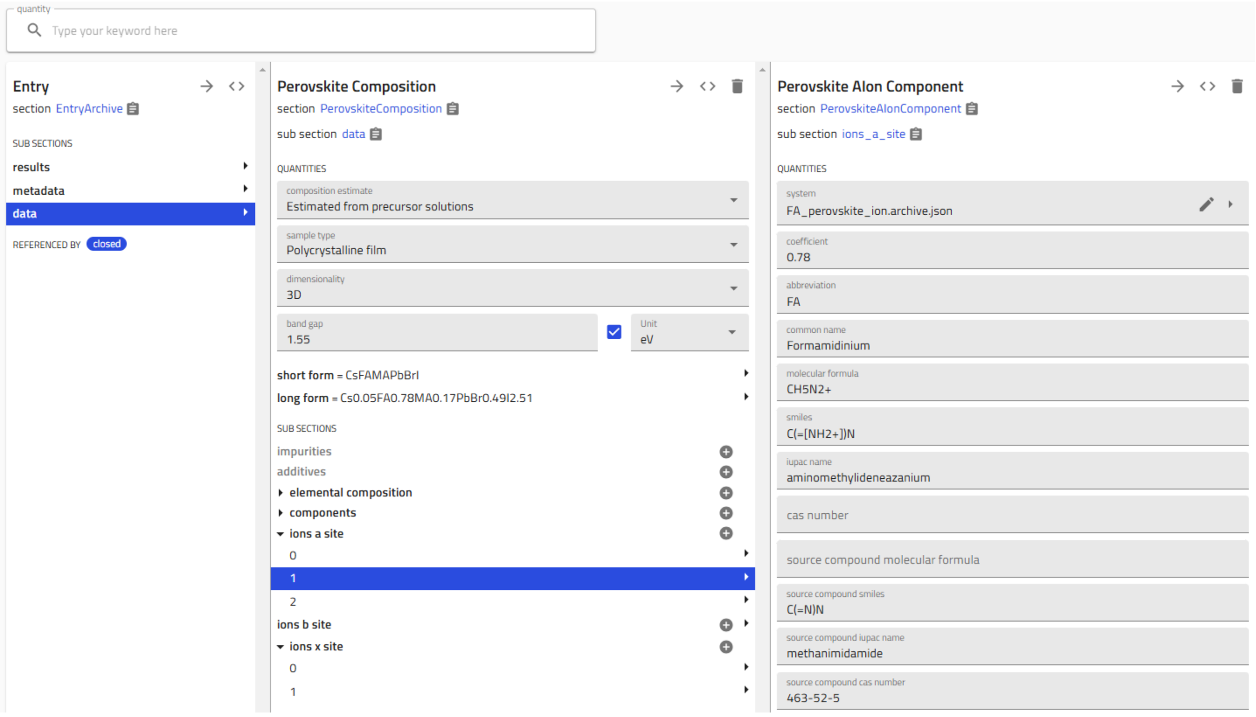

Figure S.1. NOMAD infrastructure. A graphical user interface for generating perovskite composition files, which utilise compiled data for perovskite ions for simplified workflows.

Python Utilities

As a complement to the NOMAD implementation, we have developed Python utilities that accept user input, perform a set of checks and normalisations, and generate properly formatted data files. These routines are implemented as a Python class, making them easily integrable into custom workflows.

To simplify data entry, we have also created a graphical user interface (Fig. S.2) and a Jupyter notebook demonstrating the workflows. Detailed instructions can be found in the GitHub repository at https://github.com/Jesperkemist/Perovskite_composition.

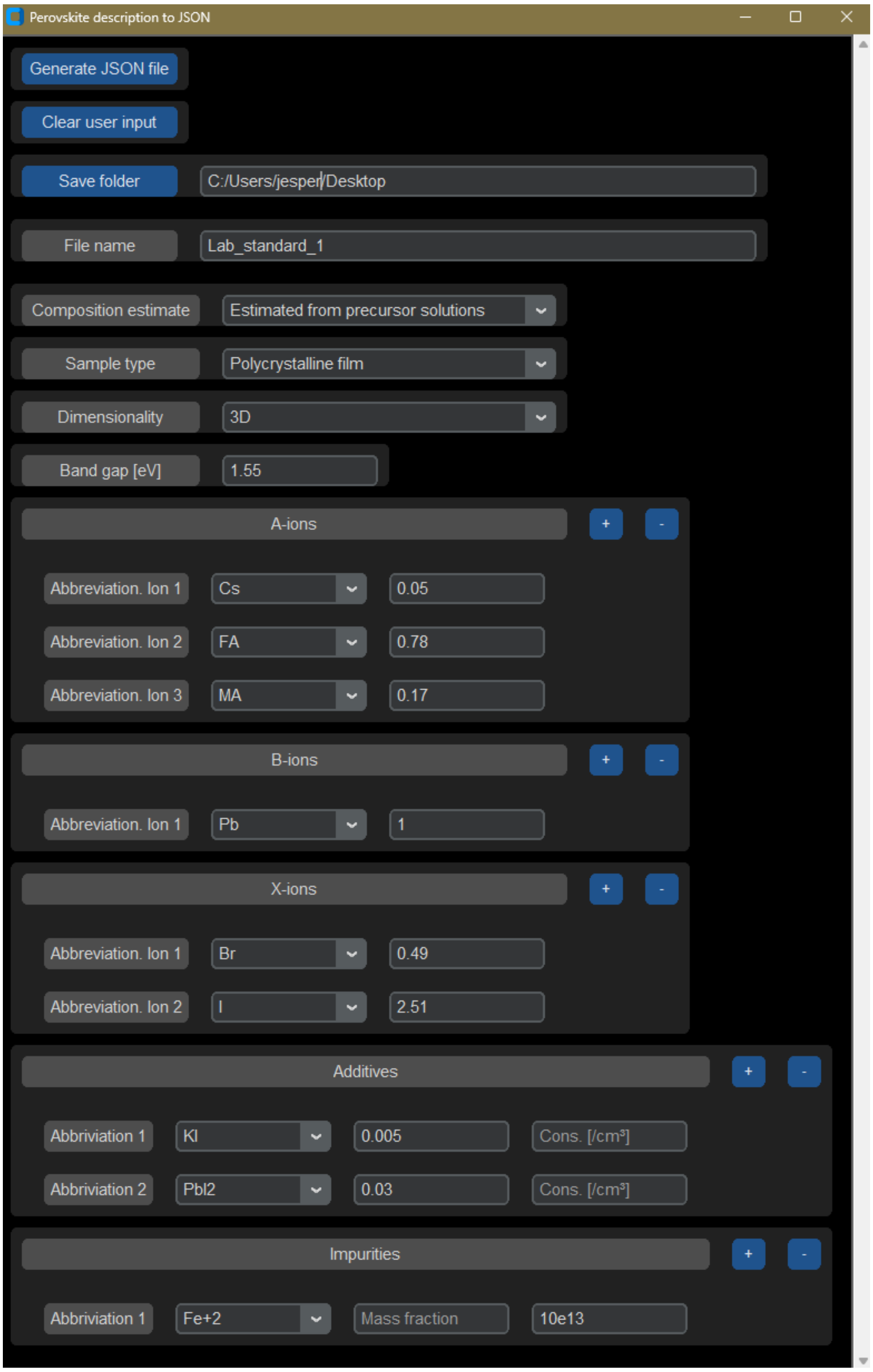

Figure S.2. A graphical user interface for generating perovskite datafiles. The resources are available as Python code. If common abbreviations are used for the ions, filling in the data fields shown in the figure is enough to generate a properly formatted perovskite composition file.
