# Supplementary material for: Towards an interoperable perovskite description or how to keep track of 300 perovskite ions
Source: Nat Commun. 2025 Sep 30;16:8725. doi: 10.1038/s41467-025-64325-x (PMC12484728; doi:10.1038/s41467-025-64325-x)
Supplement: Supplementary file 2 — Description Of Additional Supplementary File [file 41467_2025_64325_MOESM2_ESM.pdf]

Description of Additional supplementary files

Supplementary data 1

An Excel file with data for all identified perovskite ions

Supplementary data 2

A JSON validation schema for the perovskite composition

Supplementary data 3

A JSON validation schema for perovskite ions

Supplementary data 4

Example 1 of a perovskite composition files

Supplementary data 5

Example 2 of a perovskite composition files
